# Exploring the Multifaceted Biological Activities of Anthocyanins Isolated from Two Andean Berries

**DOI:** 10.3390/foods13162625

**Published:** 2024-08-21

**Authors:** Carlos Barba-Ostria, Saskya E. Carrera-Pacheco, Rebeca Gonzalez-Pastor, Johana Zuñiga-Miranda, Arianna Mayorga-Ramos, Eduardo Tejera, Linda P. Guamán

**Affiliations:** 1 Escuela de Medicina, Colegio de Ciencias de la Salud, Universidad San Francisco de Quito USFQ, Quito 170901, Ecuador; cbarbao@usfq.edu.ec; 2 Centro de Investigación Biomédica, Facultad de Ciencias de la Salud Eugenio Espejo, Universidad UTE, Quito 170147, Ecuadorrebeca.gonzalez@ute.edu.ec (R.G.-P.); johana.zuniga@ute.edu.ec (J.Z.-M.); arianna.mayorga@ute.edu.ec (A.M.-R.); 3 Bio-Cheminformatics Research Group, Universidad de Las Américas, Quito 170504, Ecuador; eduardo.tejera@udla.edu.ec

**Keywords:** anthocyanins, antioxidant activity, antimicrobial activity, antibiofilm activity, SDG 3: good health and well-being

## Abstract

Natural pigments extracted from plant species are used in foods, cosmetics, and pharmaceuticals. This study evaluates the comprehensive biological activities of anthocyanins isolated from Andean blueberry (*Vaccinium floribundum* Kunth) and Andean blackberry (*Rubus glaucus* Benth), focusing on their antimicrobial, antioxidant, antitumoral, anti-inflammatory, and hemolytic properties. Chemical characterization revealed significant anthocyanin content with complex mass spectrometric profiles indicating diverse glycosylation patterns that may influence their bioactivity. The antimicrobial assays showed that the extracts were particularly effective against Gram-positive bacteria, with minimal inhibitory concentrations (MICs) as low as 1 mg/mL for *Rubus glaucus*, indicating strong potential for therapeutic use. The antioxidant capacity of the berries was substantial, albeit slightly lower than that of ascorbic acid. The extracts also exhibited notable antitumoral activity in various cancer cell lines, showing promise as adjunctive or preventive treatments. The anti-inflammatory effects were confirmed by inhibiting nitric oxide production in macrophage cells, highlighting their potential in managing inflammatory diseases. In terms of hemolytic activity, *Rubus glaucus* exhibited dose-dependent effects, potentially attributable to anthocyanins and phenolics, while *Vaccinium floribundum* demonstrated no significant hemolytic activity, underscoring its safety. These findings suggest that anthocyanins from Andean berries possess potent biological activities, which could be leveraged for health benefits in pharmaceutical and nutraceutical applications. Further studies are needed to isolate specific bioactive compounds and investigate their synergistic effects in clinical and real-world contexts.

## 1. Introduction

Exploring natural compounds for their health-promoting properties remains a pivotal area of research within nutraceutical and pharmaceutical sciences. Among these compounds, anthocyanins, predominantly found in berries, have garnered significant attention due to their diverse biological activities [1,2]. This study focuses on anthocyanins isolated from two Andean berries: the Andean blueberry (*Vaccinium floribundum* Kunth) and the Andean blackberry (*Rubus glaucus* Benth); henceforth, these will be referred to as ‘berries’.

Berries are rich in anthocyanins and traditionally consumed in the Andes for their reputed therapeutic benefits, including antioxidant, antimicrobial, and anti-inflammatory effects [2,3,4].

Recent studies underscore the role of anthocyanins in disease prevention and health enhancement, particularly their capacity to combat oxidative stress, inflammation, and microbial infections [5,6]. The biochemical diversity of anthocyanins facilitates a wide range of cellular interactions, modulating various metabolic and physiological pathways [7,8]. However, the effectiveness of these interactions is often contingent upon the bioavailability and chemical stability of anthocyanins, which are influenced by their botanical sources and extraction methods [9,10].

In this study, we employed analytical methods to elucidate the anthocyanin profiles of these berries. Additionally, we evaluated their antimicrobial efficacy against clinically relevant bacterial strains and investigated their antioxidant capacities, aiming to correlate the presence of specific anthocyanins with observed biological effects. Such comprehensive assessments are essential for substantiating the health claims of berry-derived anthocyanins and advancing their potential therapeutic applications. Moreover, our investigation extended to assessing the anthocyanins’ antitumoral, anti-inflammatory, and hemolytic properties. This study describes the anthocyanin profiles and biological activities of *V. floribundum* Kunth and *R. glaucus* Benth berries, highlighting their potential as significant contributors to the health food and pharmaceutical sectors. The anthocyanin content and distinct biological activities of these berries underscore their importance in bioactive research and their prospective applications in combating microbial resistance.

## 2. Materials and Methods

### 2.1. Chemicals and Materials

All reagents and chemicals used in these experiments were of analytical grade or culture grade. The chemicals and reagents were sourced from Sigma Aldrich (St. Louis, MO, USA), Thermo Fisher Scientific (Miami, FL, USA), Corning (Manassas, VA, USA), and Eurobio (Les Ulis, France). Additionally, some materials were acquired from RephiLe Bioscience Ltd. (Acton, MA, USA) and Merck KGaA (Darmstadt, Germany).

### 2.2. Plant Material

Berries were sourced at a local market in Ambato, Ecuador. These fruits were selected, washed with tap water, disinfected using 100 ppm of chlorine, and freeze-dried. The samples were stored at −20 °C and subsequently used as feedstock for the experiments in this study.

### 2.3. Anthocyanin Extraction and Characterization

Berries were washed, ground, and lyophilized to obtain powdered particles. Subsequently, 10 g of the powdered material was combined with a solution containing 96% ethanol (ACS reagent, Sigma Aldrich) and 1.5 mol/L HCl (BioReagent, Sigma Aldrich) in an 85:15 *v*/*v* ratio. This mixture was placed in an oil bath, agitated, and maintained at 70 °C for 60 min to facilitate the extraction of anthocyanins. The mixture was then separated using a solid–liquid filtration method connected to a vacuum pump. The solid residue was further extracted with an additional 200 mL of the ethanol acidic solution, repeating this process three times. The liquid extracts were combined and transferred to a flask equipped with continuous agitation. The flask was connected to a rotary evaporator (R300, Buchi, Germany) operating at 70 °C to eliminate the solvent under vacuum conditions for two hours. The resulting extract was lyophilized to ensure complete desiccation, yielding the final dried product.

The dry extract was suspended in methanol–water (80:20) (15 mg/1 mL), ultrasonicated for 30 min, continuously shaken for two hours, and protected from light at room temperature. Later, samples were centrifuged for 10 min at 5000 rpm (10 °C), and the supernatant was filtered through a 0.45 μm Minisart filter (RephiLe Bioscience Ltd., Acton, MA, USA). The solid residue was re-extracted with the same volume, and supernatants were mixed. The extract was concentrated in a rotary evaporator at 30 °C, and the dry extract was stored at −20 °C until further analysis.

### 2.4. Spectrophotometric Determination of Anthocyanin

The dry extract was suspended in methanol–water (80:20) (15 mg/mL) for the determination of total anthocyanin content. The pH differential method was employed to quantify the total anthocyanin (ACY), with results expressed as milligrams of pelargonidin chloride equivalents (PgEq) per 100 g of dried weight (DW) (PgEq/g DW) [11]. The calibration curve was prepared using pelargonidin chloride (Sigma Aldrich, CAS 134-04-3).

### 2.5. HPLC-DAD Analysis of Anthocyanins

The dry extract described previously was suspended in methanol–water (80:20) (15 mg/1 mL), filtered through a 0.45 μm Minisart filter (RephiLe Bioscience Ltd., Acton, MA, USA), and analyzed using HPLC-DAD-MS/MS. The HPLC-DAD methodology was performed according to previous studies [12]. The HPLC system consisted of a Vanquish (Thermo Fisher Scientific, Waltham, MA, USA) fitted with a binary pump and DAD coupled with an LTQ-XL (Thermo Fisher Scientific, Waltham, MA, USA) controlled by Xcalibur Software version 4.3. An Accucore Vanquish C18 column (1.5 μm, 100 × 2.1 mm) (Merck KGaA, Darmstadt, Germany) thermostated at 40 °C was used as the stationary phase. In contrast, a solution of 0.1% formic acid (A) and acetonitrile (B) was used for elution. The flow rate was 0.2 mL/min. Double line detection was carried out in DAD at 280, 220, 330, and 370 nm as preferred wavelengths, and MS was operated in positive and negative ion mode. The dependent data analysis (DDA) was performed on the five most intense ions with a normalized collision energy of 35. Spectra between *m*/*z* 50 and *m*/*z* 1500 were recorded in positive and negative ionization mode. The most relevant parameters for positive and negative ionization modes are as follow: source voltage 4.5 kV, 5.0 kV, capilar temperature 275 °C, sheath gas flow of 25 psi, and capillary voltage of 18 and −21 V. Data analysis was carried out using MzMine 3.3.0. The computation of the peak area normalization was performed with MzMine using the linear normalization and total raw signal methods.

### 2.6. Antimicrobial Activity Assay

The antimicrobial efficacy of the extracts was assessed against a panel of bacterial and yeast strains. Three Gram-positive microorganisms, *Staphylococcus aureus* ATCC 25923, *Enterococcus faecalis* ATCC 29212, and *Listeria monocytogenes* ATCC 13932, along with four Gram-negative bacterial strains, *Pseudomonas aeruginosa* ATCC 27853, *Salmonella typhimurium* ATCC 14028, *Burkholderia cepacea* ATCC 25416, and *Escherichia coli* ATCC 25922, were included. Additionally, four yeast strains, *Candida albicans* ATCC 10231, *Candida krusei* ATCC 14243, *Candida glabrata* ATCC 66032, and *Candida tropicalis* ATCC 1369, were tested. All strains were obtained from the American Type Culture Collection (ATCC, Manassas, VA, USA) and maintained at −80 °C with 25% (*v*/*v*) glycerol supplementation.

Antimicrobial activity was evaluated by determining the minimum inhibitory concentration (MIC) using the agar dilution technique as described by the Clinical and Laboratory Standards Institute (CLSI) guidelines [13]. Serial dilutions of the extracts were prepared within a concentration range of 1–30 mg/mL. These dilutions were introduced into molten Mueller–Hinton agar maintained at 45 °C in a water bath. The resulting agar–extract solution mixture was thoroughly mixed and then poured into Petri dishes to solidify at room temperature.

Ciprofloxacin served as the positive control, while the negative control consisted of the microorganism suspension alone (without berry extract). Colonies isolated from a 24–48 h agar plate were used to prepare the initial inoculum, which was adjusted to a 0.5 McFarland standard, corresponding to approximately 1 × 10^8^ colony-forming units (CFU)/mL. A 1:10 dilution of the 0.5 McFarland suspension in sterile broth yielded a concentration of 10^7^ CFU/mL. Two microliters (µL) of the bacterial suspension was dispensed onto the agar, resulting in a final inoculum of approximately 10^4^ CFU per spot. The inoculated plates were allowed to stand at room temperature for 30 min to facilitate absorption, then inverted and incubated at 37 °C for 24 h. The MIC was recorded as the lowest extract concentration that inhibited bacterial growth.

### 2.7. Antioxidant Activity

Antioxidant activity was assessed using the 2,2-diphenyl-1-picrylhydrazyl (DPPH) assay, as described previously [14]. DPPH was dissolved in 100% methanol (HPLC grade) to a final concentration of 0.2 mM. Seven serial dilutions of the extract were prepared in duplicate in a 96-well microplate, with a final volume of 100 µL per well using 100% methanol. One replicate served as a blank with the addition of 100 µL of 100% methanol, while 100 µL of DPPH solution was added to the other replicate. Ascorbic acid was used as a positive control, serially diluted from a stock concentration of 50 µg/mL and prepared following the same procedure as the extracts. Samples were incubated in the dark for 40 min at room temperature, after which absorbance was measured at 515 nm using a Cytation5 multi-mode detection system (BioTek). The percentage of DPPH scavenging activity was calculated using the following formula:

$$\%\mathrm{DPPH}\;\mathrm{scavenging}=100\times \frac{\left(A_{\mathrm{sample}+\mathrm{DPPH}-}A_{\mathrm{sample}\;\mathrm{blank}}\right)}{\left(A_{\mathrm{DPPH}}-A_{\mathrm{solvent}}\right)}$$

IC_50_ values were calculated using the GraphPad Prism software version 10.2. The assay was performed at least in triplicate.

### 2.8. Antibiofilm Activity

The antibiofilm activity of the extracts was evaluated against various biofilm-forming microorganisms. *S. aureus* ATCC 25923, *E. faecalis* ATCC 29212, *L. monocytogenes* ATCC 13932, *P. aeruginosa* ATCC 9027, *B. cepacia* ATCC 25416, and the fungal strain *Candida tropicalis* ATCC 13803 were cultured in Tryptic Soy Broth medium supplemented with 1% glucose (TSB+G) overnight at 37 °C. Subsequently, microbial suspensions were prepared at a 1:100 dilution of the overnight cultures, with or without berry extracts (50 to 0.1 mg/mL). A 150 µL aliquot of each suspension was added to 96-well polystyrene plates. The plates were incubated at 37 °C under static conditions for 24 h. The medium containing free-floating microbes was removed by aspiration with a micropipette, and the wells were washed three times with phosphate-buffered saline (PBS) 1X (pH 7.2). The plates were dried for 1 h at 60 °C in a laboratory oven. The biofilms were then stained with 150 µL of 0.1% crystal violet for 20 min at room temperature, followed by three washes with PBS 1X (pH 7.2). Finally, 150 µL of 96% ethanol was added to each well for 30 min, and the optical density (OD) of the samples was measured at 570 nm. The biofilm inhibition percentage was calculated using the following formula:

$$\mathrm{Inhibitory}\;\mathrm{rate}\;\left(\%\right)=[\frac{{\mathrm{OD}}_{570\;\mathrm{nm}}\left(\mathrm{Positive}\;\mathrm{control}\right)-{\mathrm{OD}}_{570\;\mathrm{nm}}\left(\mathrm{Sample}\right)}{{\mathrm{OD}}_{570\;\mathrm{nm}}\left(\mathrm{Positive}\;\mathrm{control}\right)}]\times 100$$

### 2.9. Antitumoral Activity Assay

Four tumoral cell lines, MDA-MB-231 and MCF-7 (human breast carcinoma), HeLa (human cervical carcinoma), and THJ29T (thyroid carcinoma), along with one non-tumoral cell line, NIH3T3 (mouse NIH/Swiss embryo fibroblasts), were obtained from ATCC and cultured in Dulbecco’s Modified Eagle Medium (DMEM/F12) (Corning, Corning in Manassas, VA, USA) supplemented with 10% fetal bovine serum (FBS) (Eurobio, Les Ulis, France) and 1% penicillin/streptomycin (Thermo Fisher Scientific, Gibco, Miami, FL, USA). All cell lines were maintained at 37 °C in a humidified atmosphere with 5% CO_2_. To assess the effect of the extracts on cell proliferation, cells were seeded at a density of 1 × 10^4^ cells/well in 96-well plates. Cells were incubated for 72 h with 100 μL of the berry extracts at concentrations ranging from 0.04 to 5 mg/mL. Following the incubation period, the 3-(4,5-Dimethylthiazol-2-yl)-2,5-diphenyltetrazolium bromide (MTT) dye assay was performed following the standard procedure provided by Sigma (St. Louis, MO, USA) [15]. Briefly, 10 μL of MTT solution (5 mg/mL) was added to each well. After 1–2 h of incubation in a humidified environment, the medium was aspirated, and 50 μL of dimethylsulfoxide (DMSO) was added to dissolve the formazan crystals. The mixture was agitated for 5 min before measuring absorbance at 570 nm using a Cytation5 multi-mode detection system (BioTek, Winooski, VT, USA). Each data point was derived from quadruplicate samples, and the experiment was replicated at least four times. Dose–response curves were generated using GraphPad Prism version 10.2 (GraphPad Software, San Diego, CA, USA) to determine the concentration required to inhibit 50% of cell proliferation (IC_50_). The control group of untreated cells served as the reference for 100% cell proliferation.

### 2.10. Anti-Inflammatory Activity

RAW264.7 murine macrophage cells were obtained from ATCC and cultured in RPMI (Corning, Manassas, VA, USA) supplemented with 10% fetal bovine serum (FBS) (Eurobio, Les Ulis, France) and 1% penicillin/streptomycin (Thermo Fisher Scientific, Gibco, Miami, FL, USA). Cells were maintained at 37 °C in a humidified atmosphere with 5% CO_2_. To assess the anti-inflammatory effects of the berry extracts, RAW264.7 cells were cultured in 24-well plates at a density of 4 × 10^5^ cells per well. The macrophage cells were pretreated with the extracts at concentrations of 0.1 and 0.5 mg/mL, followed by stimulation with 1 μg/mL lipopolysaccharide (LPS). Aspirin (ASA) at 0.8 mg/mL and dexamethasone (DEX) at 0.5 μg/mL were included as controls in the assay. Nitric oxide (NO) production was measured by the intensity of color developed from the reaction between the culture medium and the Griess reagent. The concentrations of the compounds and extracts used in the assay were selected based on their IC_50_ values and did not significantly affect cell growth [16]. The assay was performed at least in triplicate.

### 2.11. Hemolytic Activity Assay

The hemolytic activity of *V. floribundum* (Vf) and *R. glaucus* (Rg) was measured following a previously described protocol [17]. Ten milliliters of defibrinated sheep blood was subjected to three consecutive washes with PBS 1X. After washing, a 1% erythrocyte suspension in PBS 1X was prepared. The erythrocyte suspension was then mixed 1:1 with berry extracts at concentrations of 10 and 50 mg/mL in PBS 1X. Positive (10% Triton X-100) and negative (PBS 1X) controls were included in the assay. The mixtures were placed in a 96-well polypropylene plate and incubated at 37 °C for 1 h. Following incubation, the mixtures were centrifuged (5 min, 1700× *g*), and the resulting supernatant was carefully transferred to a transparent flat-bottom 96-well plate for absorbance measurement at 405 nm using a Cytation 5 plate reader. Each experiment was performed with three technical replicates, and the entire protocol was repeated three times.

## 3. Results

### 3.1. Chemical Characterization

The total anthocyanin content in *R. glaucus* and *V. floribundum* was quantified, revealing 26.48 mg PgEq/gram and 51.76 mg PgEq/gram, respectively. This significant difference in anthocyanin concentration between the two fruits, as determined by the pH differential method and HPLC, reflects the overall quantity of anthocyanins rather than the number of distinct anthocyanin species. The chromatographic analysis further supports this finding, showing that *V. floribundum* has a higher total anthocyanin concentration, though this does not necessarily imply a greater diversity of anthocyanin compounds compared to *R. glaucus*.

The data presented in Table 1 summarize the identification of the most abundant peaks in *R. glaucus* and *V. floribundum*, using both positive and negative ionization modes. Table 1 details the retention times (RTs), mass-to-charge ratios ([M − H]^−^ and [M + H]^+^), and MS/MS fragmentation patterns for each peak, along with the normalized area expressed as a percentage of the total ion count. This dataset provides a comprehensive profile of the anthocyanin composition in these fruits, highlighting both differences and similarities in their anthocyanin content. The compounds were identified through spectral comparisons against the GNPS and MzCloud databases, supplemented by literature references, as outlined in Table 1. Detailed chromatographic profiles, including peak identification and retention times, are provided in the Supplementary Materials.

Figure S1 in the Supplementary Materials presents the chromatographic images, visually corroborating the findings. Both fruits exhibit a broadly similar chemical profile; however, *V. floribundum* displays a greater diversity and abundance of anthocyanins. This observation aligns with the quantified total anthocyanin content, reinforcing the reliability of the spectrometric and chromatographic data.

The higher anthocyanin content in *V. floribundum*, as compared to *R. glaucus*, may be attributed to several factors, including genetic variation [1], environmental conditions [18,19], and the developmental stages of the fruits [19]. A comparison of the anthocyanin profiles reveals that *V. floribundum* possesses a richer and more diverse array of anthocyanins, as evidenced by the greater number of peaks identified in its chromatogram. This diversity may enhance the bioactivity of *V. floribundum* [20,21], offering a more complex blend of health-promoting compounds.

**Table 1:** Anthocyanin identification in positive ionization mode in both fruits by HPLC-MS/MS.

Peak ID	RT (min)	[M − H]^−^	MS/MS	[M + H]^+^	MS/MS	Identification	*V. floribundum*	*R. glaucus*	Reference
1	1.18	191	191->111(100), 173(65), 127(20), 85(15)	193	193->147(100), 157(90), 175(25), 165(15)	Quinic acid	0.03	0.003	[20]
2	3.91			221	221->185(100), 203(30), 167(25), 95(5) 441->221(100), 185(10)	Quinic acid derivate	0.63	0.004	[17]
3	7.80	219	219->111(100), 173(95), 157(10),87(5), 191(5)	221	221->203(100), 175(30), 185(10) 203->157(100), 185(45), 175(15)	Quinic acid derivate isomer	0.06	0.35	[17]
4	10.45			177	177->131(100), 145(90), 177(40), 117(35), 103(15)	N.I	0.07	0.60	
	10.45			141	141->141(100)	N.I	0.01	0.02	
	10.93			465	465->303(100)	Delphinidin-3-pyranoside	0.40	0.004	[21]
5	11.55	447	447->285(100), 245(25), 321(20) 179(10)	449	449->287(100)	Cyanidin-3-pyranoside	2.85	0.10	[17]
	11.55			435	435->303(100)	Delphinidin-3-arabinoside	0.32	0.002	[17]
	11.97			611	611->287(100), 449(15)	Cyanidin-3-pyranoside hexoside	0.001	0.02	[17]
6	12.28	417	417->285(100), 371(40), 339(15), 299(10)	419	419->287(100)	Cyanidin-3-arabinoside	2.05	0.000	[17]
	12.27	593	593->285(100), 299(30),	595	595->287(100), 449(20)	Derivate of cyanidin 3-O-sambubioside	0.002	2.54	[22]
	12.33			727	727->287(100), 581(30), 375(10)	Cy-3-xylosylrutinoside	0.000	0.51	[22]
	12.69			433	433->271(100), 387(15)	Pelargonidin 3-glucoside	0.02	0.38	[22]
7	13.42	13.42			155	155->109(100), 127(5)	2.79	0.64	
8	13.53	345, 247	345->247(100), 157(10) 247->157(100), 201(20), 229(10), 129(10)	249	249->203(100), 231(10), 175(10) 203->157(100), 185(60), 175(5)	N.I	6.25	13.65	
9	14.67			287	287->241(100), 167(90), 185(70), 231(50), 213(45)	Cyanidin	1.42	0.02	
	14.73			557	557->287(100), 243(10)	catechin-(4-8) cyanidin	0.26	0.02	
10	20.48			575	575->299(100), 271(10)	N.I	0.10	0.07	
	20.48			277	277->203(100), 231(55), 157(5)	N.I	6.82	7.30	

### 3.2. Minimum Inhibitory Concentration

Antimicrobial susceptibility testing is critical for the effective management of pathogenic microorganisms. Additionally, determining minimum inhibitory concentrations (MICs) is fundamental in monitoring resistance development and establishing optimal pharmacodynamic dosing. The antimicrobial activity of *V. floribundum* Kunth and *R. glaucus* was evaluated against twelve clinically significant microbial strains.

The in vitro susceptibility tests indicated that *R. glaucus* extracts exhibited potent inhibitory effects against the Gram-positive bacteria *E. faecalis*, *S. aureus*, and *L. monocytogenes*, with MIC values ranging between 1 and 1.2 mg/mL. In contrast, *V. floribundum* displayed higher MIC values, ranging from 2.1 to 2.5 mg/mL. For Gram-negative bacteria, the MIC values for both berries ranged between 8 and 18 mg/mL.

Table 2 presents the MICs for each microbial strain as determined by the agar dilution method, highlighting distinct differences between the two berries. Overall, *R. glaucus* demonstrated greater efficacy compared to *V. floribundum* across all tested bacteria, yielding the lowest MICs. Both Gram-positive and Gram-negative strains exhibited susceptibility to the berry extracts, with MICs varying according to the berry species and bacterial strain. Notably, MICs were lower for Gram-positive strains compared to Gram-negative strains in both berries. The lowest MIC was observed for *R. glaucus* against *E. faecalis* at 1 mg/mL, whereas the highest MIC was 18 mg/mL for *V. floribundum* against *Salmonella typhimurium*.

The relatively lower inhibitory activity observed in Gram-negative bacteria is likely attributable to the presence of an outer lipopolysaccharide membrane that encases the bacterial cell wall. The absence of this membrane in Gram-positive bacteria may facilitate greater permeability of bioactive phytochemicals, resulting in more pronounced bacterial inhibition [22,23]. The MIC values for the positive control, ciprofloxacin, were consistent with previously reported values, falling within one- or two-fold dilutions of the expected MIC [24]. Proanthocyanidins present in berries, consisting of units of (−)-epicatechin and/or (+)-catechin linked by type A and B interflavanic bonds, are recognized for their antibacterial efficacy against both Gram-negative and Gram-positive bacteria. These compounds interact with bacterial membranes, increasing their permeability, which leads to membrane puncturing, disintegration, and ultimately, the death of the bacterial cells [25].

The table presents the retention times (RTs), mass-to-charge ratios ([M − H]^−^ and [M + H]^+^), and MS/MS fragmentation patterns for the most abundant peaks identified in *R. glaucus* and *V. floribundum* under both positive and negative ionization modes. The values presented in columns *V. floribundum* and *R. glaucus* correspond to the normalized peak area expressed in percent. Identification was based on spectral comparisons against the GNPS and MzCloud databases, supplemented by literature references. Detailed chromatographic images are provided in the Supplementary Materials. The results obtained in our research on the antifungal activity of anthocyanins are particularly noteworthy, considering the limited evidence available in the scientific literature. To date, only a few studies have evaluated the antimicrobial activity of anthocyanins, primarily using inhibition zone measurements in millimeters. This approach is not directly comparable to the MIC determinations we performed, which are expressed in mg/mL [26]. The lack of standardized methodologies complicates direct comparisons. However, a recent study reported MIC values for anthocyanins ranging from 100 to 200 mg/mL, consistent with our findings, thereby validating the relevance of our methodologies and results [27]. This study provides a valuable reference point, underscoring the significance of our contributions to the antifungal evaluation of anthocyanins.

While the exact mechanism of action for the antifungal activity of anthocyanins against *Candida* strains remains unclear, the disruption of cell membrane integrity by anthocyanins has been previously documented. The potential mechanisms underlying this activity likely involve both membrane interactions and intracellular effects of anthocyanin functional groups. The microbicidal potential of fruits and other berries containing polyphenols, including anthocyanins, is likely due to multiple modes of action, given the diversity of molecules involved—ranging from weak organic acids to phenolic acids and glycosides in various chemical forms [28,29].

Overall, our data suggest that *R. glaucus* holds significant potential as a source of effective antimicrobial agents for use in the pharmaceutical and food industries. However, further analyses are required to determine whether the antimicrobial activity of the extracts is attributable to a single bioactive compound or the complementary, synergistic, and additive effects of multiple phytochemicals. This phenomenon will likely depend on various factors, including geographical and environmental conditions [29].

### 3.3. Biofilm Inhibition Assay

**Figure 1:**
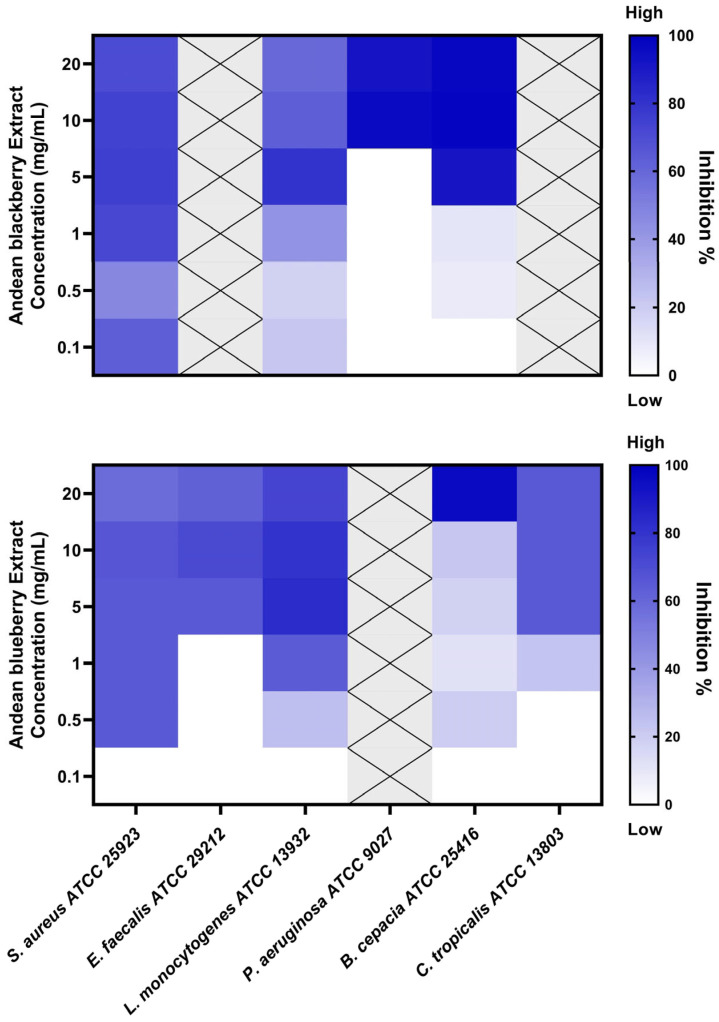
Inhibition of biofilm-forming strains by *R. glaucus* and *V. floribundum* extracts, evaluated by crystal violet (CV) staining. The heatmap displays the biofilm inhibition percentage, calculated over the corresponding strain’s growth control. Dark blue indicates the highest inhibition percentage, while light blue/white indicates a low/null inhibition percentage. Grey crossed-out squares indicate that the microorganism strains were excluded from the MBIC_50_ analysis after initial screening due to the absence of inhibition at all evaluated concentrations.

The extracts were tested to assess their biofilm inhibition activity against biofilm-forming microorganisms, including *S. aureus*, *E. faecalis*, *L. monocytogenes*, *P. aeruginosa*, *B. cepacia*, and *C. tropicalis*. The *R. glaucus* extract exhibited biofilm inhibition potential against four strains, while the *V. floribundum* extract demonstrated activity against five of the tested microorganisms, as illustrated in Figure 1.

The minimum biofilm-inhibiting concentrations with 50% or more inhibition effectiveness (MBIC_50_) are presented in Table 3. MBIC values exceeding 20 mg/mL were designated as “non-active” (NA). The *R. glaucus* extract displayed the lowest MBIC value against *S. aureus*, achieving 72% inhibition at a concentration of 1 mg/mL. In contrast, the lowest MBIC_50_ value for the *V. floribundum* extract was 0.5 mg/mL, resulting in a 64% inhibition of *S. aureus* biofilm.

Our study revealed that the *V. floribundum* extract exhibited the lowest MBIC at 0.5 mg/mL against *S. aureus*, with a corresponding inhibition percentage of 64% (Table 3). Similar findings have been reported in the literature, with one study documenting an MBIC of 0.5 mg/mL and a 68% inhibition percentage in methicillin-sensitive *S. aureus* (MSSA). Additionally, the same study found that this extract concentration could inhibit methicillin-resistant *S. aureus* (MRSA) biofilms by 49% [30], suggesting that future antibiofilm research should include the evaluation of *V. floribundum* extract against antibiotic-resistant *S. aureus* strains.

Liu et al. (2021) also investigated the effects of *Brightwell* blueberry extracts on *L. monocytogenes* biofilms, reporting an MBIC of 2 mg/mL (~84% inhibition rate), which is slightly higher than the MBIC obtained in our study [19]. It is noteworthy that Liu et al. employed an optimized anthocyanin concentration protocol involving purification with AB-8 resin and freeze-drying, as described by Gao and colleagues [31]. Liu et al. (2021) suggest that concentrated anthocyanin-rich extracts may exhibit enhanced antimicrobial effects compared to crude extracts [19]. The difference in MBIC values between Liu et al.’s findings and our study could be attributed to the use of this anthocyanin concentration protocol and slight variations in the phytochemical compositions of the fruits from different regions.

Our research is the first to assess the biofilm inhibition potential of blueberry and blackberry extracts against *C. tropicalis*. The *V. floribundum* extract demonstrated an MBIC of 5 mg/mL, resulting in a 64% inhibition of *C. tropicalis* biofilm. In contrast, the *R. glaucus* extract showed no biofilm-inhibitory effects on *C. tropicalis* at the tested concentrations. The literature on berry extracts and biofilm inhibition is sparse; one study evaluated the impact of Clery strawberry extracts against *C. albicans*, reporting significant antibiofilm potential [32]. Further research is required to explore the biofilm inhibition potential of berry extracts against *Candida* spp. and to elucidate the biological activities of blackberry extracts.

Wu et al. (2022) investigated the antimicrobial effects of blackberry extracts against periodontopathogens, which are known for their biofilm-forming capabilities [18]. Similar to Liu et al. (2021), Wu et al., employed an AB-8 resin macroporous-based purification process to concentrate anthocyanins from blackberry extracts. They reported an MBIC of 2 mg/mL and an inhibition rate of ~93% against *L. monocytogenes* biofilm. The use of anthocyanin concentration protocols in these studies could explain the enhanced biofilm inhibition activity observed, contrasting with the results obtained using the crude extract in our study.

Implementing anthocyanin concentration protocols in future research could significantly improve the biofilm inhibition efficacy of berry extracts. Moreover, studies focusing on the fractionation and purification of specific active compounds within berry extracts could provide valuable insights into their individual biological activities [33]. Additionally, co-administration of blackberry extracts with antibiotics, such as methicillin, has demonstrated promising results in other studies [34].

### 3.4. Antioxidant Activity

Both berries exhibited very similar antioxidant potential; however, this potential was 4- to 4.4-fold lower than that of the ascorbic acid control (Table 4).

Our findings are consistent with those reported by Alarcón-Barrera et al. (2018), who demonstrated that *V. floribundum* possesses higher DPPH scavenging activity than *R. glaucus* [35].

In our study, *V. floribundum* Kunth demonstrated an IC50 of 21.77 µg/mL in the DPPH assay, while Prencipe et al. (2014) reported an IC50 of 0.694 µg/mL for acidified methanolic extracts of the same berries [36]. This discrepancy may be attributed to the different extraction methods employed, as Prencipe et al. used maceration with EtOAc followed by dynamic maceration with HCl in MeOH, which could have enhanced the extraction efficiency and antioxidant activity. Furthermore, Guevara-Terán (2022) highlighted the importance of considering the maturity stage of the plant when evaluating its antioxidant activity, noting that samples collected at high altitudes (3641 m.a.s.l) exhibited significant variations in antioxidant activity depending on their maturity stage [17]. Similarly, Samaniego (2020) observed that as Andean blackberries matured, their phytochemical components diminished, leading to reduced antioxidant activity [37].

Various methodologies, including ABTS, the modified TEAC assay, oxygen radical absorbance capacity (ORAC), and FRAC, have been employed to assess the antioxidant potential of *V. floribundum* and *R. glaucus* [4,23,38]. These studies underscore the significance of these berries as natural sources of anthocyanins and their potential health benefits as antioxidants.

### 3.5. Antitumoral Activity

The antitumoral activity of the berry extracts was evaluated using the MTT assay to assess cell proliferation. Treatment with both extracts resulted in significant dose-dependent inhibition of cell proliferation, particularly for *R. glaucus*, with IC_50_ values ranging from 1.22 to 3.69 mg/mL, showing the highest effect against HeLa cells. Notably, the IC_50_ values for *R. glaucus* were lower than those for *V. floribundum* in all tested cell lines, except for MDA-MB-231 cells (Table 5). Comparable IC_50_ values have been reported for blackberry extracts in studies involving colon tumoral cells [39], where the authors highlighted the potential of blackberry extracts as a source of bioactive compounds beneficial in treating diseases associated with oxidative stress. Additionally, the blueberry extract demonstrated a dose-dependent inhibitory effect on cell proliferation across various tumor cell lines, reducing cell adhesion and inhibiting the migration of MDA-MB-231 and PC-3 tumor cells [40].

Both berries contain anthocyanins, known for their potent antioxidant activity, which may inhibit carcinogen activation and DNA damage [41]. Furthermore, anthocyanins are implicated in the suppression of cancer cell growth through the modulation of cell signaling pathways and cell cycle regulators [42,43]. Similarly, research indicates that blueberry anthocyanins hold therapeutic potential in protecting liver cells from oxidative stress [44].

On the other hand, both extracts exhibited antiproliferative effects on non-tumoral NIH3T3 cells, with IC_50_ values of 2.22 mg/mL for *R. glaucus* and 2.60 mg/mL for *V. floribundum*. These findings underscore the necessity for further studies to evaluate the safety and efficacy of these extracts in cancer therapy.

### 3.6. Anti-Inflamatory Activity

In the context of our study, the modulation of inflammatory responses, primarily through the inhibition of nitric oxide (NO) production in RAW264.7 cells, is a crucial aspect in evaluating the anti-inflammatory activity of the berry extracts. Previous studies have demonstrated that blueberry extracts can suppress the production of pro-inflammatory cytokines and chemokines, whereas blackberry extracts are known to inhibit inflammatory mediators such as TNF-α, IL-6, IL-1β, COX-2, and iNOS, indicating their potential anti-inflammatory properties [45,46].

**Figure 2:**
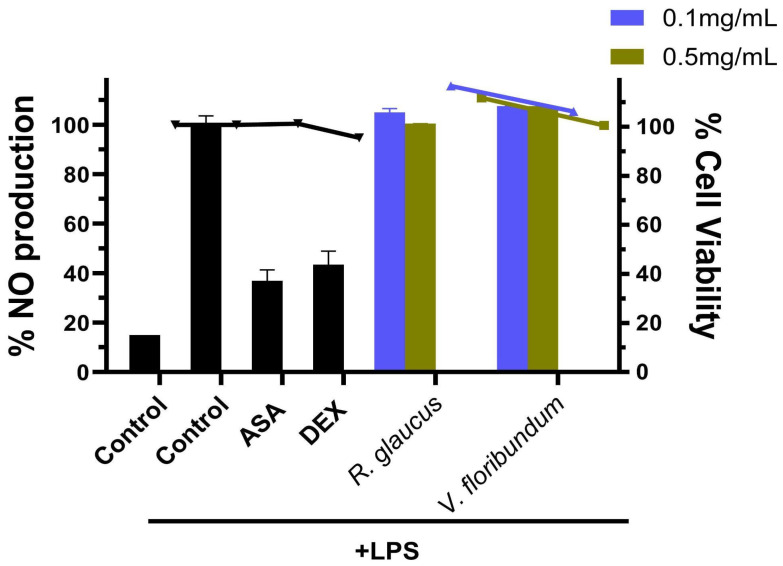
Anti-inflammatory activity *of R. glaucus* and *V. floribundum* extracts. RAW264.7 cells were pretreated with the sample compounds and then stimulated with LPS. The percentage of NO production was calculated using cells treated with LPS only (control + LPS) as 100% NO production (right Y axis). Aspirin (ASA) and dexamethasone (DEX) were used as positive controls, and cells were incubated only with cell media as negative controls (control). Bars represent the percentage of NO production. Dots and lines represent the % cell viability for each corresponding dataset (left Y axis)—black: *R. glaucus*; blue: *Vaccinium floribundum* extract.

In our experimental setup, RAW264.7 cells were pretreated with the berry extracts before being stimulated with LPS to induce an inflammatory response. The NO levels in the culture media were subsequently measured using the Griess method. As expected, LPS exposure activated the inflammatory response in RAW264.7 cells, leading to a significant increase in NO production after 18 h of LPS stimulation (Figure 2). Pretreatment with aspirin and dexamethasone before LPS challenge effectively reduced NO production to 36.9% and 43.3%, respectively. Notably, NO levels in the LPS-stimulated group were significantly higher compared to the control group (media only).

However, our results indicate that pretreatment with 0.1–0.5 mg/mL of the berry extracts did not significantly suppress NO production in LPS-induced RAW264.7 cells. This lack of significant suppression suggests that the anti-inflammatory activity of these extracts may vary, potentially influenced by factors such as the fruit’s variety, ripeness, and processing conditions [47].

### 3.7. Hemolytic Activity

Hemolysis, characterized by the rupture of red blood cells and subsequent release of hemoglobin, serves as a critical indicator of cytotoxicity. The hemolytic activity (HA) of aqueous extracts from *R. glaucus* (labeled Rg) and *V. floribundum* (labeled Vf) was assessed following a previously described protocol [15]. The analysis revealed significant differences in the cytotoxic potential of these extracts, which can be attributed to the distinct profiles of bioactive compounds present in each. The results of the hemolytic assay indicate that *R. glaucus* exhibits dose-dependent hemolytic activity, whereas *V. floribundum* shows negligible hemolytic activity (Table 6).

In this study, *R. glaucus* at 10 mg/mL demonstrated a hemolytic activity of 6.3%, which increased to 10.2% at 50 mg/mL, indicating a significant dose-dependent rise in hemolytic activity (Table 6). This higher hemolytic activity observed in *R. glaucus* is likely due to its bioactive compound profile, particularly the presence of delphinidin-3-pyranoside and cyanidin-3-pyranoside, identified in the extract (Table 1). Previous research has shown that anthocyanins and other phenolic compounds found in berries can exhibit cytotoxic effects, including hemolysis [48,49]. These studies suggest that the specific anthocyanins present in *R. glaucus* contribute to its hemolytic activity.

In contrast, *V. floribundum* at 10 mg/mL exhibited no hemolytic activity, indicating that the aqueous extracts of Vf are non-hemolytic at the tested concentration. *V. floribundum* contains anthocyanins such as cyanidin-3-arabinoside and delphinidin-3-pyranoside, but at lower concentrations, which may contribute to its lack of hemolytic activity.

The contrasting hemolytic activities observed between *R. glaucus* and *V. floribundum* underscore species-specific differences in bioactive compound profiles. The higher hemolytic activity in *R. glaucus* suggests the presence of anthocyanins and phenolics capable of disrupting cell membranes. On the other hand, the absence of hemolytic activity in *V. floribundum* implies a composition that is either devoid of cytotoxic compounds or enriched with protective compounds that prevent membrane disruption. These findings carry significant implications for the potential therapeutic applications of these extracts.

## 4. Conclusions

This study evaluated the bioactive potential of *V. floribundum* Kunth and *R. glaucus* extracts, demonstrating their potential as natural antimicrobial, antioxidant, and antitumor agents with significant health benefits. The chemical characterization revealed a rich content of anthocyanins, as evidenced by their complex mass spectrometric profiles, which suggest a robust framework for their bioactivity. Notably, the antimicrobial activity assays highlighted that *R. glaucus* extracts were particularly effective, exhibiting low MIC values against Gram-positive bacteria such as *E. faecalis*, *S. aureus*, and *L. monocytogenes*. These findings underscore the potential of *R. glaucus* as a natural antimicrobial source suitable for applications in both the pharmaceutical and food industries.

However, the extracts demonstrated less activity against Gram-negative bacteria, likely due to the protective outer membrane of these bacteria, which may hinder the penetration of bioactive compounds. This observation suggests the need for further studies to enhance the efficacy of these extracts against Gram-negative pathogens, potentially through formulation improvements or combination therapies.

Moreover, both berry extracts displayed significant antioxidant activity, although slightly less than that of standard ascorbic acid, indicating their potential as natural antioxidants to enhance food shelf life and safety. The antioxidant capacity, combined with antimicrobial effects, provides a compelling case for using these extracts in food preservation and as therapeutic agents. Regarding the antitumor assays, the extracts showed dose-dependent inhibition of cell proliferation across various cancer cell lines, with the most pronounced effects observed in HeLa cells. *R. glaucus* exhibited lower IC_50_ values compared to *V. floribundum* for most cell lines, indicating greater potency. The extracts also displayed antiproliferative effects on non-tumoral NIH3T3 cells, highlighting the need for further research to assess the safety and therapeutic potential of these compounds.

Additionally, our findings revealed no significant anti-inflammatory activity, suggesting that variations in the fruit’s properties may influence its biological effects.

The hemolytic activity assay provided valuable insights into the cytotoxic potential of the berry extracts. *R. glaucus* exhibited dose-dependent hemolytic activity, likely due to its specific anthocyanin and phenolic content, while *V. floribundum* showed no significant hemolytic activity, corroborating its reputation as a safe, antioxidant-rich berry. The total anthocyanin content and specific profiles of bioactive compounds play a crucial role in these effects. Future research should focus on isolating and characterizing the specific compounds responsible for these effects, further elucidating their mechanisms of action and potential therapeutic applications. Additionally, in vivo studies are necessary to confirm the safety and efficacy of these extracts in clinical settings.

In conclusion, our findings suggest that the studied berry extracts, particularly those from *R. glaucus*, offer promising potential for development as products that could contribute to health promotion and disease prevention. Future studies focusing on mechanistic pathways, dosage optimization, and clinical trials are necessary to fully integrate these natural products into the pharmaceutical and food sectors.

## Figures and Tables

**Table 2:** Minimum inhibitory concentrations (MICs) expressed in mg/mL of *V. floribundum* Kunth and *R. glaucus* extracts against bacterial strains of clinical importance. Ciprofloxacin (CP), expressed in µg/mL, was used as a positive control.

	Strain	*R. glaucus*	*V. floribundum*
		MIC mg/mL	MIC mg/mL
Gram-positive	*Staphylococcus aureus* ATCC 25923	1.2	2.1
	*Enterococcus faecalis* ATCC 29212	1.0	2.5
	*Listeria monocytogenes* ATCC 13932	1.2	2.2
Gram-negative	*Pseudomonas aeruginosa* ATCC 27853	8	12
	*Salmonella typhimurium* ATCC 14028	10	16
	*Burkholderia cepacea* ATCC 25416	10	14
	*Escherichia coli* ATCC 25922	12	18
Yeast	*Candida krusei* ATCC 14243	110	150
	*Candida glabrata* ATCC 66032	150	180
	*Candida tropicalis* ATCC 1369	120	100
	*Candida albicans* ATCC 10231	100	100

**Table 3:** Minimum biofilm-inhibiting concentration of six susceptible microbial strains treated with *R. glaucus* or *V. floribundum* extracts.

Strains	*R. glaucus* Extract		*V. floribundum* Extract	
	MBIC_50_ (mg/mL)	Inhibition Percentage	MBIC_50_ (mg/mL)	Inhibition Percentage
*Staphylococcus aureus* ATCC 25923	1	72 ± 6.6%	0.5	64 ± 7.1%
*Enterococcus faecalis* ATCC 29212	NA	NA	5	63 ± 12.2%
*Listeria monocytogenes* ATCC 13932	5	80 ± 16.7%	1	63 ± 5.7%
*Pseudomonas aeruginosa* ATCC 9027	10	96 ± 2.0%	NA	NA
*Burkholderia cepacia* ATCC 25416	5	91 ± 9.1%	20	96 ± 3.5%
*Candida tropicalis* ATCC 13803	NA	NA	5	64 ± 12.9%

NA: non-active at the tested concentrations.

**Table 4:** IC_50_ values corresponding to the DPPH assay expressed in µg/mL of the extract or ascorbic acid.

IC_50_	*Rubus glaucus*	*Vaccinium floribundum*	Control
µg/mL	24.13 ± 3.73	21.77 ± 3.15	5.47 ± 0.30

**Table 5:** Inhibitory concentration values (IC_50_) of extracts against tumor and non-tumor cell lines at 72 h.

Cell Lines	*R. glaucus* (mg/mL)	*V. floribundum* (mg/mL)
MDA-MB-231	3.69 ± 0.60	2.31 ± 0.23
MCF-7	3.33 ± 0.76	>5.00
HeLa	1.40 ± 0.31	>5.00
THJ29T	2.38 ± 0.75	>5.00
NIH3T3	2.22 ± 0.20	2.60 ± 0.90

**Table 6:** Hemolytic activity of extracts.

	% Hemolysis
C−	0 ± 0.3
C+	100.0 ± 1.4
Rg 10 mg/mL	6.3 ± 0.5
Rg 50 mg/mL	10.2 ± 3
Vf 10 mg/mL	0 ± 1.3
Vf 50 mg/mL	1.3 ± 2.5

## Data Availability

The data presented in this study are openly available in FigShare at https://doi.org/10.6084/m9.figshare.26321551.v1 (accessed on 19 August 2024).

## Supplementary Materials

The following supporting information can be downloaded at: https://www.mdpi.com/article/10.3390/foods13162625/s1, Figure S1: Chromatogram representation of both extracts covering total ion count, UV range in 210–500 nm and UV range in 400–500 nm, Figure S2: Proposed rupture mechanism for the parent ion at 575 *m*/*z*, Table S1: Identification of most abundant peaks in positive and negative ionization mode in both fruits.
