# Supplementary material for: Exploring the Multifaceted Biological Activities of Anthocyanins Isolated from Two Andean Berries
Source: Foods. 2024 Aug 21;13(16):2625. doi: 10.3390/foods13162625 (PMC11353912; doi:10.3390/foods13162625)
Supplement: Supplementary file 1 [file foods-13-02625-s001.zip › foods-3136758-supplementary.pdf]

Supplementary Material

In Figure S1 we shown the chromatogram obtained in both extracts. We also showed the same chromatogram covering two UV ranges: 210-500 nm and 400-500 nm. We can notice that both chromatograms are very similar but the UV profile is quite different specially in the UV-vis range.

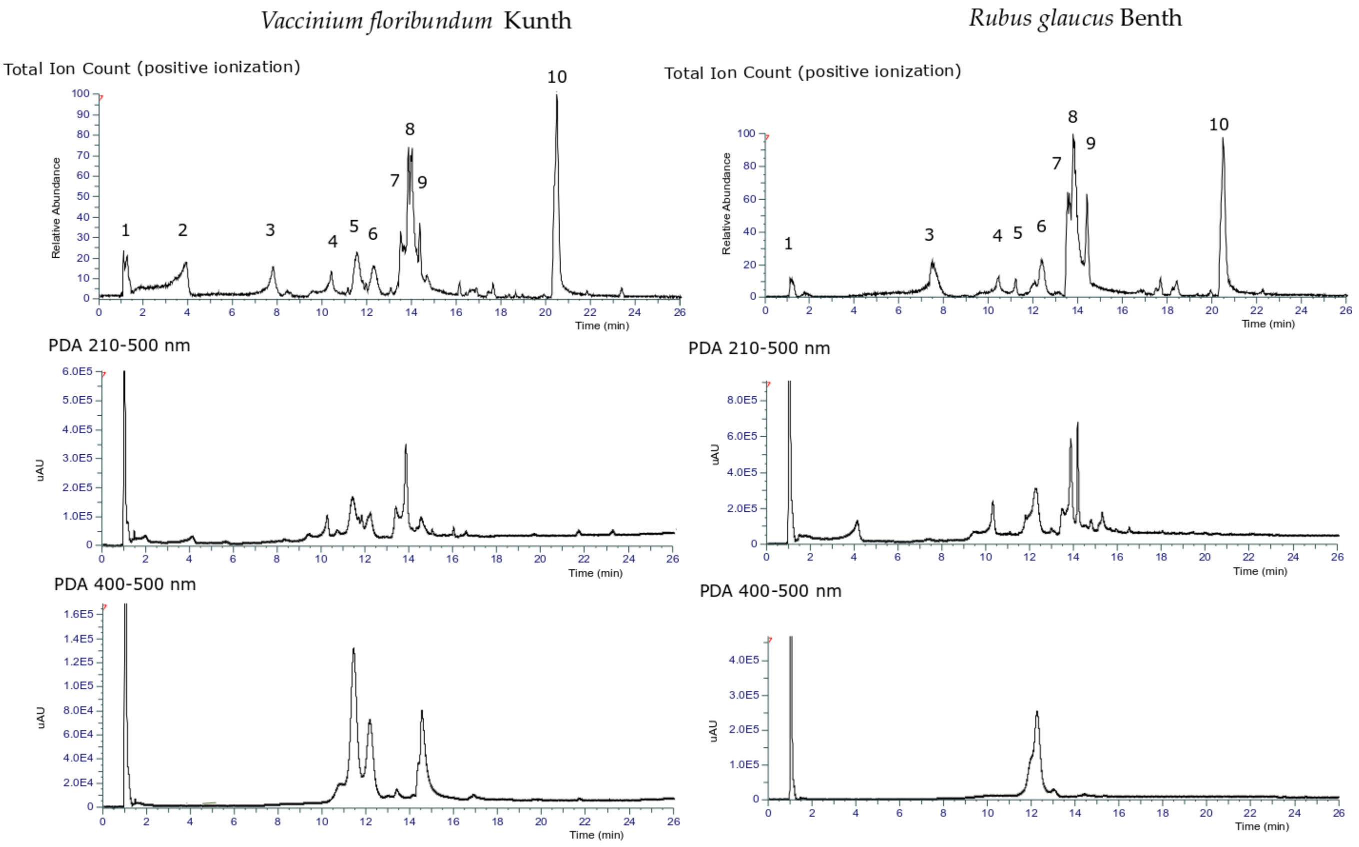

Figure S1. Chromatogram representation of both extracts covering total ion count, UV range in 210-500 nm and UV range in 400-500 nm.

The UV range in 400-500 nm is an UV-vis range used for anthocyanin detection. In this case it is notice that Andean blueberry (Vaccinium floribundum Kunth) has an increased anthocyanin range that Andean blackberry (Rubus glaucus Benth). It is consistent with the information presented in Table S1 and also in the total content of anthocyanin.

Table S1. Identification of most abundant peaks in positive and negative ionization mode in both fruits.

Peak ID	RT (min)	[M-H]-	MS/MS	[M+H]+	MS/MS	Identification	Andean Blueberry	Andean Blackberry	Refference
1	1.18	191	191-> 111(100), 173(65), 127(20), 85(15)	193	193->147(100), 157(90), 175(25), 165(15)	Quinic acid	X	X	(Aita et al. 2021) [20]
2	3.91			221	221->185(100), 203(30), 167(25), 95(5) 441->221(100), 185(10)	Quinic acid derivate	X		(Guevara-Terán et al. 2022) [17]
3	7.80	219	219->111(100), 173(95), 157(10),87(5), 191(5)	221	221->203(100), 175(30), 185(10) 203->157(100), 185(45), 175(15)	Quinic acid derivate isomer	X	X	(Guevara-Terán et al. 2022) [17]
4	10.45			177	177->131(100), 145(90), 177(40), 117(35), 103(15)	N.I		X	
	10.45			141	141->141(100)	N.I		X	
	10.93			465	465->303(100)	Delphinidin-3-pyranoside	X		(Stein-Chisholm et al. 2017) [21]
5	11.55	447	447->285(100), 245(25), 321(20) 179(10	449	449->287(100)	Cyanidin-3-pyranoside	X	X	(Guevara-Terán et al. 2022) [17]
	11.55			435	435->303(100)	Delphinidin-3-arabinoside	low intensity		(Guevara-Terán et al. 2022) [17]
	11.97			611	611->287(100), 449(15)	Cyanidin-3-pyranoside hexoside	low intensity		(Guevara-Terán et al. 2022) [17]

6	12.28	417	417->285(100), 371(40), 339(15), 299(10)	419	419->287(100)	Cyanidin-3-arabinoside	X		(Guevara-Terán et al. 2022) [17]
	12.27	593	593->285(100), 299(30),	595	595->287(100), 449(20)	Derivate of cyanidin 3-O-sambubioside		X	(Alcalde-Eon et al. 2016) [22]
	12.33			727	727->287(100), 581(30), 375(10)	Cy-3-xylosylrutinoside		X	(Alcalde-Eon et al. 2016) [22]
	12.69			433	433-> 271(100), 387(15)	Pelargonidin 3-glucoside	low intensity	X	(Alcalde-Eon et al. 2016) [22]
7	13.42	13.42			155	155-> 109(100), 127(5)			
8	13.53	345, 247	345->247(100), 157(10) 247->157(100), 201(20), 229(10), 129(10)	249	249->203(100), 231(10), 175(10) 203-> 157(100), 185(60), 175(5)	N.I	X	X	
9	14.67			287	287->241(100), 167(90), 185(70), 231(50), 213(45)	Cyanidin	Low intensity		
	14.73			557	557->287(100), 243(10)	catechin-(4-8) cyanidin	X	low intensity	
10	20.48			575	575-> 299(100), 271(10)	N.I			
	20.48			277	277->203(100), 231(55), 157(5)	N.I			

The parent mass at m/z 727 (peak 6) is fragmented into 581 m/z with a neutral loss of 146 uma (possible a rhamnose unit [https://www.ncbi.nlm.nih.gov/pmc/articles/PMC6245369/]). The ion at 581 is possible fragmented into 287 m/z that we think it is cyanidin and a loss of hexose (162 uma) and pentose (132 uma) explaining the neutral loss of 294 uma. This neutral loss is more likely sambubioside as discussed in [https://doi.org/10.1016/j.phytochem.2016.04.004]. Similarly, the parent mass of 611 (peak 5, RT=11.97 min), loss 162 uma (hexose) with further fragmentation to cyanidin pyranoside (m/z 449).

Around 13 min, we have several important ions, specially 155 m/z, 249 m/z, 287 m/z and 557 m/z (peaks 7, 8 and 9). The most intense ions are 155 m/z (RT=13.42) and 249 m/z (RT=13.53). These molecules were not possible to identified. The loss of 36 uma from 249 to 203 and from 155 to 109 could be associated with carboxylic acid loss. The rupture in 155 is similar to benzoic acid derivate but the RT is too high for this type of molecule. Similarly, the ions at RT=20.48 (Peak 10) were not identified. These ions were included in the table because it is important to provide the full information for further researchers. The identification of peak 9 have a score of 81.5 % in the comparison of the MS2 with MzCloud database. This annotation is consistent with the peak detected at this retention time in the UV-vis chromatogram (Figure S1)

In previous publicationssimilar masses of 557 and 287 and even 243 (Peak 9) had been reported as catechin derivate of cyanidin. However, our parent mass at 557 is only consistent if the [M-H2O]+ adduct is assumed. The mechanism proposed (Figure S2) would explain our masses but also can be explained if the catechin –OH is actually missing. However, considering that the catechin- (4-8) cyanidin is present in several fruits and plants we tentative assign this annotation.

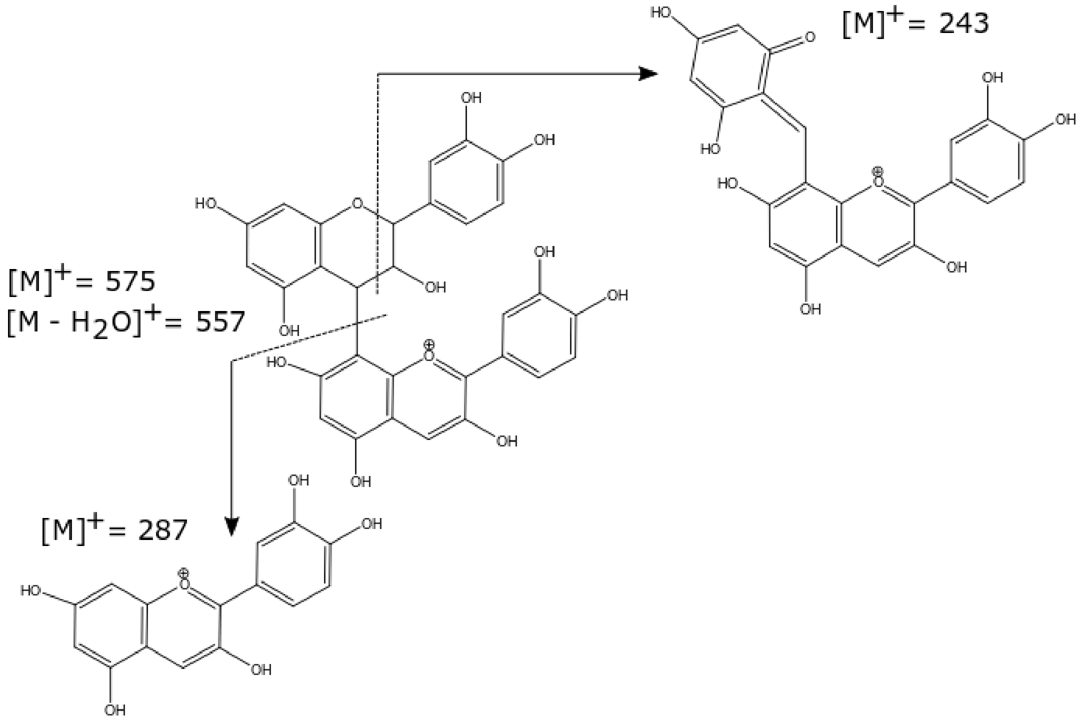

Figure S2. Proposed rupture mechanism for the parent ion at 575 m/z.

References:

17. Guevara-Terán, M.; Padilla-Arias, K.; Beltrán-Novoa, A.; González-Paramás, A.M.; Giampieri, F.; Battino, M.; Vásquez-Castillo, W.; Fernandez-Soto, P.; Tejera, E.; Alvarez-Suarez, J.M. Influence of Altitudes and Development Stages on the Chemical Composition, Antioxidant, and Antimicrobial Capacity of the Wild Andean Blueberry (Vaccinium floribundum Kunth). Molecules 2022, 27, 7525. https://doi.org/10.3390/molecules27217525.

20. Aita, S.; Capriotti, A.; Cavaliere, C.; Cerrato, A.; Giannelli Moneta, B.; Montone, C.; Piovesana, S.; Laganà, A. Andean Blueberry of the Genus Disterigma: A High-Resolution Mass Spectrometric Approach for the Comprehensive Characterization of Phenolic Compounds. Separations 2021, 8, 58. https://doi.org/10.3390/separations8050058.

21. Stein-Chisholm, R.; Beaulieu, J.; Grimm, C.; Lloyd, S. LC–MS/MS and UPLC–UV Evaluation of Anthocyanins and Anthocya-nidins during Rabbiteye Blueberry Juice Processing. Beverages 2017, 3, 56. https://doi.org/10.3390/beverages3040056.

22. Alcalde-Eon, C.; García-Estévez, I.; Rivas-Gonzalo, J.C.; Rodríguez de la Cruz, D.; Escribano-Bailón, M.T. Anthocyanins of the anthers as chemotaxonomic markers in the genus Populus L. Differentiation between Populus nigra, Populus alba and Populus tremula. Phytochemistry 2016, 128, 35–49. https://doi.org/10.1016/j.phytochem.2016.04.004.
